# Glucagon-like Peptide 1, Glucose-Dependent Insulinotropic Polypeptide, and Glucagon Receptor Agonists in Metabolic Dysfunction-Associated Steatotic Liver Disease: Novel Medication in New Liver Disease Nomenclature

**DOI:** 10.3390/ijms25073832

**Published:** 2024-03-29

**Authors:** Lampros G. Chrysavgis, Spyridon Kazanas, Konstantina Bafa, Sophia Rozani, Maria-Evangelia Koloutsou, Evangelos Cholongitas

**Affiliations:** 1 First Department of Internal Medicine, Medical School, National and Kapodistrian University of Athens, General Hospital Laiko, 115 27 Athens, Greece; lchrisaugis@gmail.com (L.G.C.); sp.kazanas@gmail.com (S.K.); konstantina.mpaf@gmail.com (K.B.); sofrozan@gmail.com (S.R.); 2 First Department of Propaedeutic Internal Medicine, Medical School, National and Kapodistrian University of Athens, General Hospital Laiko, 115 27 Athens, Greece; mkoloutsou@yahoo.com

**Keywords:** metabolic dysfunction-associated steatotic liver disease, glucagon-like peptide-1 (GLP-1), glucose-dependent insulinotropic polypeptide (GIP), glucagon receptor (GCGR), type 2 diabetes mellitus

## Abstract

Glucagon-like peptide 1 (GLP-1) and glucose-dependent insulinotropic polypeptide (GIP) are incretins that regulate postprandial glucose regulation, stimulating insulin secretion from pancreatic β-cells in response to food ingestion. Modified GLP-1 receptor agonists (GLP-1RAs) are being administered for the treatment of obesity and type 2 diabetes mellitus (T2DM). Strongly related to those disorders, metabolic dysfunction-associated steatotic liver disease (MASLD), especially its aggressive form, defined as metabolic dysfunction-associated steatohepatitis (MASH), is a major healthcare burden associated with high morbidity and extrahepatic complications. GLP-1RAs have been explored in MASH patients with evident improvement in liver dysfunction enzymes, glycemic control, and weight loss. Importantly, the combination of GLP-1RAs with GIP and/or glucagon RAs may be even more effective via synergistic mechanisms in amelioration of metabolic, biochemical, and histological parameters of MASLD but also has a beneficial impact on MASLD-related complications. In this current review, we aim to provide an overview of incretins’ physiology, action, and signaling. Furthermore, we provide insight into the key pathophysiological mechanisms through which they impact MASLD aspects, as well as we analyze clinical data from human interventional studies. Finally, we discuss the current challenges and future perspectives pertinent to this growing area of research and clinical medicine.

## 1. Introduction

On a global scale, metabolic dysfunction-associated steatotic liver disease (MASLD), the latest term for the definition of the disease previously known as non-alcoholic fatty liver disease (NAFLD) [1], is exhibiting a growing trajectory, affecting approximately one-third of the general population, and is now acknowledged as the most widespread chronic liver disease worldwide [2]. MASLD is strongly associated with metabolic disorders, namely type 2 diabetes mellitus (T2DM), dyslipidemia, and obesity. The reported prevalence of MASLD in overweight, obese, and T2DM patients are estimated as high as 70%, 75%, and 55%, respectively [3,4]. MASLD encompasses patients with hepatic steatosis and at least one of five metabolic risk factors, namely central obesity, hypertension, pre-diabetes or T2DM, hypertriglyceridemia, and low HDL cholesterol. Similarly, the advanced form of the disease is now called metabolic dysfunction-associated steatohepatitis (MASH) and has replaced the term non-alcoholic steatohepatitis (NASH) [5]. MASH is characterized by the additional emergence of hepatocellular damage, lobular inflammation, and hepatocyte ballooning and can be accompanied by fibrosis [5]. MASH can result in advanced fibrosis and, ultimately, cirrhosis, whereby bands of fibrous septa lead to the formation of cirrhotic nodules, as well as the development of hepatocellular carcinoma (HCC) [6,7]. The diagnosis of MASLD requires the exclusion of secondary causes of liver steatosis, such as alcoholic liver disease (ALD), chronic hepatatis C (HCV), steatogenic medication, or any other disorder possibly affecting the hepatic parenchyma [8,9]. The current recommendation for disease management mostly focuses on lifestyle interventions. These include increased physical activity with aerobic exercise along with exercise with resistance, weight loss, and stricter adherence to a Mediterranean diet [5,10,11]. However, adherence to dietary and lifestyle restrictions is challenging enough, as only 50% of MASLD patients can achieve and maintain the predefined weight loss targets in the long term. Despite the high prevalence of the disease and the vast scientific effort globally, there are currently no Food and Drug Administration (FDA) or European Medicines Agency (EMA)-approved medications for MASLD. However, notably, over the decades, several drugs have been evaluated for the treatment of MASLD, each of them aiming at one or more of the following pathophysiological aspects: insulin resistance and lipid metabolism, inflammation and immune activation, cell death, lipotoxicity, oxidative stress, and fibrogenesis [12]. Evidence from the scientific research worldwide suggests that a promising therapeutic agent for the disease comprises the incretin analogs, the most well-studied of which are the glucagon-like peptide-1 receptor agonists (GLP-1RAs) [13].

Incretin hormones are gut peptides released post-nutrient intake that triggers insulin secretion from pancreatic beta cells [14]. Glucose-dependent insulinotropic polypeptide (GIP) and glucagon-like peptide-1 (GLP-1) are recognized as incretin hormones responsible for the incretin effect, a two- to three-fold higher insulin secretory response to oral compared with intravenous glucose administration, accounting for as much as 70% of postprandial insulin secretion [15]. This effect is diminished or absent in individuals with T2DM [16]. Agonists for incretin receptors have already shown efficacy for the treatment of T2DM [17] while they concurrently exert clinically meaningful benefits for the cardiovascular and renal systems [18]. Moreover, GLP-1RAs, especially their combination with GIP receptor agonists or even triple agonists against GLP-1, GIP, and glucagon (GCG), have generated a lot of interest among the scientific community as novel effective glucose and body weight lowering agents [19,20]. Indeed, a recent meta-analysis highlighted that tirzepatide, a dual GLP-1 and GIP receptor agonist (RA), provoked significant dose-dependent amelioration in glycemic control and body weight lowering in comparison to both placebo and GLP-1Ras, such as semaglutide or dulaglutide, or basal insulin regimens [21]. To this end, those agents may be an appealing therapeutic opportunity for MASLD, especially among patients with co-existing T2DM and/or obesity [22,23].

In this current review, we summarize the current literature dealing with the efficacy of incretin RAs on MASLD and metabolic parameters related to MASLD based on animal studies. Furthermore, we discuss the current published randomized controlled trials (RCTs) of GLP-1RA on MASLD patients, as well as present the studies evaluating combined incretin agonism for MASLD or aspects of MASLD. Finally, we identify the current challenges and the open questions and issues to be addressed in the near future.

## 2. Incretin Hormones

### 2.1. Glucagon-like Peptide-1 (GLP-1) Agonism

**Figure 1:**
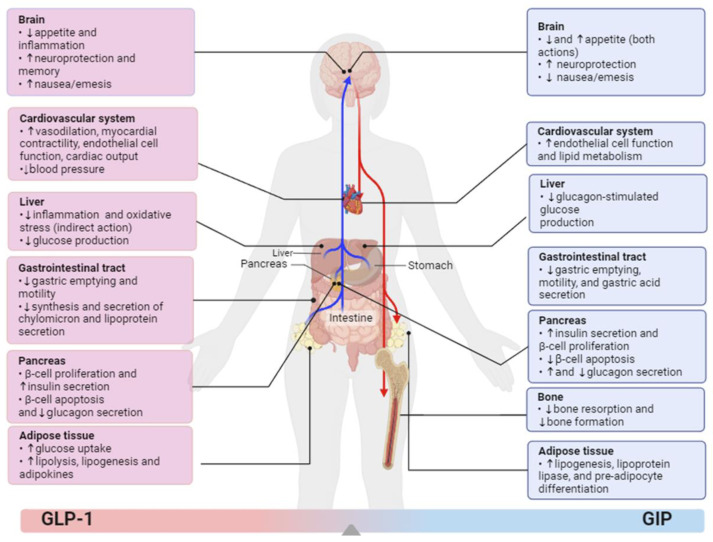
Pleiotropic effects and actions of glucagon-like peptide-1 (GLP-1) (on the left side in gradient red) and glucose-dependent insulinotropic polypeptide (GIP) (on the right side in gradient blue) in different tissues and organs. GIP, glucose-dependent insulinotropic peptide; GLP-1, glucagon-like peptide-1.

GLP-1 stands as a pivotal gastrointestinal peptide hormone, originating from the conversion of proglucagon (chromosome 2) via the enzyme prohormone convertase 1/3 (PC1/3), comprising 30 amino acids, and is excreted by specialized enteroendocrine L cells, which are located in the lower intestine [24,25]. GLP-1 is secreted throughout the 24hr at reduced basal amount in the fasting and interprandial state, while its secretion increases two–three-fold upon meal ingestion [26]. Notably, GLP-1 has a short half-life of approximately 2 min since it is rapidly metabolized by the dipeptidyl peptidase-4, and due to that, a minimal proportion estimated at 10% of gastrointestinal secreted GLP-1 reaches the systemic circulation [25]. GLP-1 exhibits well-established and recognized multidimensional biological effects within the realm of clinical medicine (Figure 1).

These effects are orchestrated through the binding of GLP-1 to its receptors (GLP1Rs), which belong to the family of G protein-coupled receptors (GPCRs), and they are present in various organs, including the brain, heart, muscles, pancreatic islets, gastrointestinal tract, and kidneys [27,28,29]. GLP-1 substantially increases insulin secretion, fostering pancreatic β-cell proliferation and enhancing β-cell survival [24,25]. Simultaneously, GLP-1 exerts inhibitory action in glucagon secretion, presumably being mediated by a direct effect on pancreatic alpha cells, therefore leading to a substantial reduction in glucagon release, diminished gluconeogenesis, and augmented hepatic storage of glucose as glycogen [24,25]. Nonetheless, that inhibitory effect can also be mediated by the paracrine action of high levels of somatostatin and insulin from neighboring delta and beta cells, respectively [24,25]. Beyond its impact on the pancreas, GLP-1 facilitates higher glucose uptake by muscle cells and enhances both glucose uptake and lipolysis in adipocytes. Notably, GLP-1 also extends its influence on cardiovascular dynamics, elevating myocardium contractility and contributing to its cardioprotective effects [30]. Furthermore, GLP-1 plays a dual role in the feeling of satiety both by suppressing gastrointestinal motility and the production of gastric acid and by its secretion from neurons in the brainstem, leading to a reduction in the sensation of hunger, food consumption, and as a result, in body weight [31]. More precisely, GLP-1RAs are internalized in neurons expressing proopiomelanocortin (POMC) and cocaine- and amphetamine-regulated transcript (CART) and suppress the activity of Agouti-related peptide (AgPR) and Neuropeptide Y (NPY) co-expressing neurons via GABA-dependent signaling in the arcuate nucleus (ARC). That action leads to the inhibition of meal initiation, as well as provoking meal termination in the lateral parabrachial nucleus [32,33], thus facilitating weight loss. Notably, GLP-1RAs suppress gastric emptying via both inhibiting peristalsis in the stomach and stimulating tonic contractions in the pyloric region, resulting in increased fasting and postprandial gastric volumes and, therefore, reduced glucose levels [34]. That finding was highlighted in patients with type 1 diabetes since exogenous GLP-1 administration led to lower fasting glycemia, as evaluated by the reduced calculated isoglycemic meal-related insulin requirement, mainly via decreasing glucagon and somatostatin concentration [35,36]. Therefore, GLP-1 administration can substantially reduce blood glucose levels regardless of the engagement of pancreatic β cells and insulin secretion. Of interest, not all GLP-1RAs exert the same effect on appetite suppression and bodyweight regulation. For instance, albiglutide has a weaker impact on lowering body weight, whereas semaglutide has more pronounced effects; despite their glucose-lowering effects being similar, a finding indicated the discrepancy in the effects on body weight [31]. Finally, GLP-1 is indirectly implicated in liver metabolism by alleviating postprandial glycemia and via that diminishing of the liver’s propensity for synthesizing new fatty acids, thus leading to a reduction in the activation of the carbohydrate response element binding protein (ChREBP) and suppression of de novo lipogenesis [37].

### 2.2. Glucose-Dependent Insulinotropic Polypeptide (GIP) Agonism

Emerging evidence has suggested that dual incretin agonism with GLP-1 and glucose-dependent insulinotropic polypeptide (GIP) could be more effective in the amelioration of MASLD [38,39]. GIP consists of 42 amino acids and is synthesized from the prohormone precursor known as pro-GIP, encoded by the genetic material on chromosome 17 [27]. That is mediated by the enzymatic action of prohormone convertase (PC) 1/3 and secreted by enteroendocrine K-cells, which are primarily located in the duodenum and proximal jejunum of the small intestine [27]. GIP exerts its physiological effects through the interaction with the GIP receptor (GIPR), which is a member of the secretin-vasoactive intestinal peptide receptor family and is widely distributed in numerous organs and tissues throughout the human body (Figure 1) [40,41,42]. GIP signaling facilitates pancreatic insulin secretion via binding to its receptor and promotes glucose-provoked insulin secretion via both cAMP-protein kinase A and EPAC2 molecular pathways [43]. However, in euglycemic or hypoglycemic states, GIP has a null impact on pancreatic insulin secretion but stimulates glucagon release, thereby contributing to normoglycemia [44,45]. In general, GIP boosts weight loss, improves the response to insulin, and modulates lipid metabolism. The sensitivity of GIPR agonism in adipose tissue has been suggested to improve the lipid-buffering capability of white adipose tissue (WAT). More specifically, via its receptors’ expression in WAT, it enhances the ability of adipocytes to effectively clear dietary triglycerides (TAGs), preventing their accumulation and ectopic fat deposition [46]. This promotes the favorable storage of excess fat while restraining storage in ectopic sites, such as the muscle or liver [47]. Effective adipose tissue storage can delay the onset of insulin resistance linked with obesity [48]. This leads to a plausible hypothesis that GIPR agonism in adipose tissue could potentially yield beneficial effects on insulin sensitivity. Impaired GIP signaling has been correlated with obesity and T2DM [49]; however, the exact pathophysiological mechanisms have not been fully elucidated. GIP can increase the activity of lipoprotein lipase (LPL), thus enhancing the clearance of triglycerides. Moreover, it stimulates the lipogenesis and differentiation of preadipocytes and reduces triglyceride levels after a lipid load [47] by promoting GLUT4 plasma membrane expression and the uptake of glucose by muscle and adipose tissues [38,39]. Thus, GIP can induce energy accumulation in adipocytes and can increase the anabolic action of insulin in the adipose tissue, effects that are indirectly beneficial for the liver.

### 2.3. Glucagon (GCG) Agonism

Glucagon (GCG) is a 29-amino acid peptide hormone [50,51] encoded like GLP-1 by the proglucagon gene and is secreted by pancreatic alpha cells in response to hypoglycemia, prolonged fasting, and exercise [52,53]. GCG acts mainly in the liver to raise serum glucose levels by glycogenolysis or gluconeogenesis, actions facilitated via GCG’s binding with the cognate 7-transmembrane G-protein coupled receptor, which provokes an intracellular signal [54,55]. However, GCG receptors (GCGR) are found, to a lesser extent than in the liver, in the brain, kidney, adipose tissue and gastrointestinal tract [53,56]. GCG agonism increases energy expenditure by promoting hepatic glycogenolysis in the acute state and by increasing sympathetic tone, which promotes the expansion of brown adipose tissue and browning of WAT [55]. Notably, GCGR agonism provokes a reduction in total food intake, an action that is dependent on ARC-localized CaMKKβ sensitivity [57] and CGGR-expressing hepatic vagal nerve afferents to the hypothalamus [58,59]. The administration of GCG can induce, independent of GCGR-provoked glucose production, enhanced insulin secretion through pancreatic β-cells via the activation of the GLP-1R in the postprandial state in a paracrine manner [60,61]. As a result, despite the provisional hyperglycemia, GCGR agonism leads to direct beneficial effects on satiety, energy expenditure, and, ultimately, obesity [55,57]. We shall point out that besides anti-obesogenic effects, the CGCR agonism in the liver also provokes amelioration in lipid metabolism [62], hepatic ureagenesis [63], and changes in the circulating profile of several amino acids [64,65].

#### Literature Search

We reviewed the current literature from the inception of the idea of this current review until December 2023. For our scope, we used the “PubMed” database, and we included only studies written in the English language. We used the following search terms: “Non-alcoholic fatty liver disease” OR “NAFLD” OR “Metabolic dysfunction-associated steatotic liver disease” OR “MASLD” OR “metabolic dysfunction-associated fatty liver disease” OR “MAFLD” AND “glucagon-like peptide-1” OR “GLP-1” OR “Glucose-dependent insulinotropic polypeptide” OR “GIP” OR “glucagon” OR “GCG” OR “dual incretin agonism” OR “triple incretin agonism”. We incorporated the principal pathophysiological mechanisms as described by animal studies. Furthermore, we included all human RCTs that evaluated GLP-1RAs on MASLD patients. Moreover, we presented the RCTs evaluating double or triple incretin agonism in patients with MASLD or on aspects of MASLD in non-MASLD cohorts. Finally, the references of the research articles were scrutinized for relevant studies.

## 3. The Beneficial Effect of Incretin Agonists on Aspects of MASLD/MASH; Evidence from Mouse Studies 

### 3.1. GLP-1 Signaling

**Figure 2:**
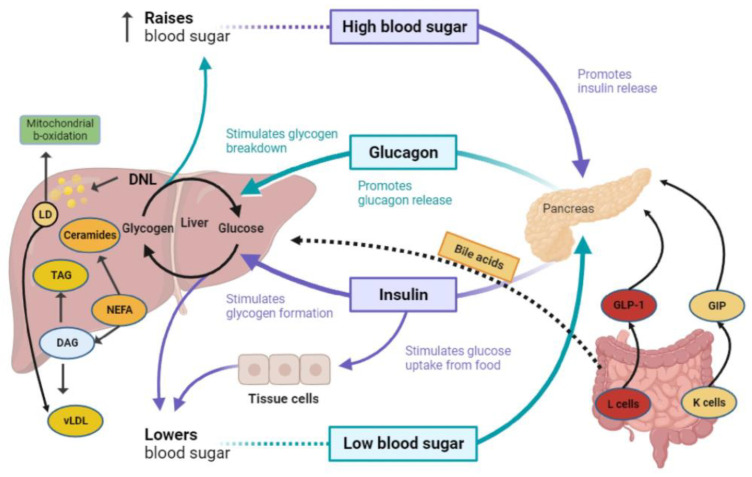
Schematic representation of hepatic glucose and lipid metabolism, along with the impact of insulin and endogenous incretin hormones on the triangle interaction between liver, pancreas, and intestine, key tissues influencing metabolic dysfunction-associated steatotic liver disease (MASLD). DAG, diacylglycerol; DNL, de novo lipogenesis; ER, endoplasmic reticulum; GIP, glucose-dependent insulinotropic peptide; GLP-1: Glucagon-like peptide-1; GLP-1RA, Glucagon-like peptide-1 receptor agonist; LD, lipid droplet NEFA, non-esterified fatty acids; TAG, triacylglycerol; VLDL, very low-density lipoprotein.

GLP-1RAs have already shown efficacy in the amelioration of hepatic steatosis, liver dysfunction enzymes, and lipid and glycemic profiles (Figure 2) [22,66]. Semaglutide administration reduced liver damage in the high-lipid diet-induced MASLD mouse model, as indicated by the reduction in serum ALT and aspartate aminotransferase (AST), low-density lipoprotein (LDL), and triglycerides levels [67]. In a Gubra-Amylin NASH (GAN) diet-induced obese (DIO) mouse model, the administration of semaglutide led to significantly reduced food intake, progressive weight loss, improved aminotransferase levels, and reduced hypercholesterolemia compared with untreated mice [68]. Moreover, semaglutide improved NAS and the expression of several extracellular matrix-related genes but, notably, did not induce the regression of fibrosis [68]. However, semaglutide inhibited the upregulation of pro-inflammatory factors, namely tumor necrosis factor-alpha (TNF-α), interleukin-6 (IL-6) and IL-1β, and malondialdehyde, a major marker of oxidative stress, thereby alleviating liver damage and inflammation [69]. Besides semaglutide, liraglutide attenuated the gene expression of the lipogenic genes acetyl-CoA carboxylase (ACC) and fatty acid synthase (FAS) while mitigating the expression of pro-inflammatory cytokines or transcription factor, including TNF-α and nuclear factor κ-light chain-enhancer of activated B cell (NF-κB) in high-fat diet (HFD) mice with ApoE and adiponectin (Acrp30) knockdown background, thus preventing MASH progression [69]. Consistent with that, Chiu et al. showed that treatment with liraglutide- and metformin-induced weight loss, reduced liver/body weight ratio, and ameliorated steatosis, liver injury, and MASH activity in a methionine/choline-deficient (MCD) diet-fed C57BL/6JNarl mouse model [70]. Of note, GLP-1 analogs induce the polarization of macrophages toward the M2 phenotype and, via that, induce the expression of anti-inflammatory molecules such as IL-10, CD163, and CD204 while concurrently reducing c-c motif chemokine ligand 2 (CCL-2) expression [71].

#### 3.1.1. Contribution of GIP to GLP-1 Signaling

Along this line, experimental mice overexpressing GIP were characterized by a reduction in diet-induced obesity and steatosis [72,73]. Moreover, GIP infusion increased glucose uptake from subcutaneous abdominal adipose tissue in patients with type 1 diabetes mellitus [72]. This, therefore, led to the increased adipose deposition of triglycerides—most likely through increased blood flow to adipose tissue. On the contrary, GIP knockout mice fed an HFD had decreased steatosis and inflammation, evaluated by lower levels of IL-6, indicating that GIP may promote lipid deposition and inhibition and that signaling could protect from that [73].

Of note, pharmacological treatment with GLP-1/GIP in a mice model of MASH seemed to further reduce steatosis, inflammation, and potentially, fibrosis and liver dysfunction enzymes, as well as ameliorating lipid and glycemic profile, compared with GLP-1 and GIP mono-agonists. More precisely, in a (DIO) mouse model, treatment with dual GLP-1/GIP RAs led to decreased fasting glucose and insulin and improved Homeostatic Model Assessment for Insulin Resistance (HOMA-IR) levels, as well as lower cholesterol, LDL, and apolipoprotein levels [74], in both male and female DIO mice compared with GLP-1 or GIP mono-agonist-treated mice [74,75]. Moreover, dual treatment reduced liver fat content (LFC) and liver weight and downregulated the hepatic expression of genes regulating cholesterol and bile acid synthesis. Other studies confirmed that the combined agonistic action of GLP-1/GIP RAs [72,76] can reduce steatosis by downregulating the hepatic expression of lipogenic genes, including the transcription factor sterol regulatory element-binding protein 1 (Srebf1), 3-hydroxy-3-methylglutaryl-CoA reductase, cytochrome P450 family 27 subfamily A member 1 (Cyp27a1), and cytochrome P450 family 7 subfamily B member 1 (Cyp7b1) [76]. Ma et al. confirmed those findings since they showed that a 28-day treatment with an oral dual GLP-1/GIP RA in mice, which were fed an HFD, resulted in lower animal food consumption and reduced body weight, as well as lower fasting blood glucose, total serum cholesterol, non-esterified free fatty acids (NEFA), and LDL cholesterol levels. It also significantly ameliorated glucose tolerance and the pancreatic β/α cell ratio, as well as decreasing the area of liver fibrosis [77]. With respect to the effect on body weight, upon GIP injection into the brain or peripherally, GIP activated neurons in the hypothalamus [78] and targeted (DREADD-mediated) the activation of hypothalamic GIPR neurons, which reduces food intake in mice [79]. Validating the metabolic significance of central GIPR signaling, the absence of neuronal GIPR results in mice showing resistance to GIP-induced weight loss. Additionally, the combined action of GIPR and GLP-1R is more effective in inducing weight loss compared with selective GLP-1R activation [78]. Suggested mechanisms for this phenomenon involve the stimulation of hypothalamic neurons that inhibit food intake and the activation of hindbrain neurons that produce an anti-emetic effect [80].

#### 3.1.2. Contribution of GCG to GLP-1 Signaling

The impact of GCG receptor engagement along with GLP-1 signaling was evaluated in DIO C57BL/6 mice, which were administered cotadutide, a dual GLP-1/glucagon receptor agonist (GCGRA) [38,81]. After four weeks, compared with baseline, it was observed that cotadutide controlled body weight gain, glucose intolerance, insulin resistance, and reduced liver fat accumulation and levels of triacylglycerol, total cholesterol, ALT, and AST [81]. In addition, cotadutide induced mitochondrial turnover and enhanced mitochondrial function, the disruption of which has been directly associated with MASH pathogenesis [82]. In this case, the increased number of labeled mitochondria within the labeled lysosomes in hepatocytes treated with cotadutide and in control cells appeared to increase the number of mitochondria and the metabolic processes within them [83]. By this mechanism, cotadutide alleviated fibrosis to a greater extent than liraglutide or obeticholic acid, despite the dose adjustment to achieve similar weight loss in these two murine models of MASH [83]. More recently, an important study by Boland et al. highlighted that cotadutide induced greater body weight loss and decreased foot intake while improving glucose control via GLP-1 mediating signaling [83]. Concurrently, treatment with cotadutide led to reduction of hepatic steatosis and ameliorated mitochondrial turnover and function in a direct GCG-dependent mechanism of action, as observed in the DIO mouse model [83]. In addition, cotadutide resolved the hepatic fibrosis stage and reduced the serum expression of C3M, a neo-epitope fragment of type III collagen cleaved during degradation, as well as circulating levels of P4NP7S, an internal epitope derived from the basement membrane collagen in ob/ob AMLN MASH mice [83]. Concurrently, cotadutide substantially suppressed inflammation, as indicated by the reduced CD68 scoring, in ob/ob AMLN MASH mice. Importantly, by applying transcriptomic analysis, authors have demonstrated that after cotadutide treatment, the hepatocyte transcript for tumor necrosis factor-alpha (TNF-α) signaling, nuclear factor κ-light chain-enhancer of activated B cell (NFκB) pathway, IL-12, IL-17, IL-4, and type 1 and type 2 helper T cells, as well as Transforming growth factor beta (TGF-b) and IL-6/Jak/Stat3, have been altered [83]. Other authors indicated that mice that were fed HFD and were treated with G49, a GLP-1/GCG RA, showed the amelioration of MASH, as evaluated by reduced inflammation, steatosis, oxidative stress, and hepatocyte apoptosis, as well as increased mitochondrial biogenesis [84]. Moreover, similar to the previous studies, dual agonist therapy was associated with enhanced gluconeogenesis and reduced glucose use via the pentose phosphate cycle and oxidative metabolism [84]. Notably, G49 administration led to increased liver regeneration even in mice initially fed an HFD and then treated with that dual RA [84]. Kannt et al. also evaluated the potential synergistic effect of different incretin RA in a murine model of MASH [85]. They demonstrated that the combination of either GCG or GIP RA with GLP-1RA led to greater weight loss, liver triglyceride reduction, and amelioration of NAS as compared to GCG or GIP mono-agonism [85]. Notably, both dual GLP-1/GCG RA and triple incretin combination significantly reduced NAS compared with high-dose liraglutide despite both interventions being adjusted to promote similar weight loss [85]. More recently, in a DIO-MASH mouse model mice, ALT-801, a dual GLP-1/GCG RA, was head-to-head compared to semaglutide monotherapy and elafibranor, a peroxisome proliferator-activated receptor, PPAR-α/δ, agonist, in a DIO mouse model of MASH [86]. ALT-801 significantly reduced body weight and serum levels of aminotransferases and total cholesterol, along with a greater decrease in steatosis in comparison to semaglutide and elafibranor. More importantly, ALT-801 ameliorated NAS score vs. both active controls, as well as inducing a greater reduction in the inflammation marker galectin-3 compared with elafibranor [86]. Consistently, TB001, a dual GLP-1/GCG RA with a higher affinity toward the latter receptor, retarded the progression of liver fibrosis in various mice models with significant selectivity, potency, and extended half-life, as well as low toxicity [87]. In addition, TB001 administration led to dose-dependent reduced liver injury and collagen accumulation, as well as the decreased activation of hepatic stellate cells via the mitigation of TGF-b expression and the downregulation of Smad signaling pathways [87]. Furthermore, TB001 attenuated liver fibrosis by inhibiting the downstream activation of the pro-inflammatory NF-kappa-B inhibitor alpha (NFκB/IKBα) pathways and blocking the c-Jun N-terminal kinase (JNK)-dependent induction of hepatocyte apoptosis [87]. Therefore, TB001 could be a promising treatment agent for hepatic fibrosis.

## 4. GLP-1 in MASLD and MASH: Randomized Clinical Trials

The number of RCTs that investigate the potential beneficial effect of GLP-1 receptor agonists on patients with MASLD/MAH is of great interest. In Table 1, we present a summary of the currently available, placebo-controlled, and active-controlled RCTs that evaluated agents with GLP-1 receptor agonists’ activity to treat MASLD or MASH. A total of 13 published RCTs are included, and the specific agents used are liraglutide (n = 6), exenatide (n = 2), dulaglutide (n = 1), and semaglutide (n = 4). Some of them exclusively enrolled patients with pre-existing T2DM (n = 7), while others recruited patients with or without T2DM (n = 6) [88,89,90,91,92,93,94,95,96,97,98,99,100].

### 4.1. Liraglutide

Liraglutide is currently the most well-studied GLP-1RA in patients with MASLD or MASH. In a UK-originated RCT, 52 overweight patients with biopsy-confirmed MASH were randomly assigned to receive either liraglutide 1.8 mg once daily or placebo for 48 weeks. At the end of the study, 39% of the patients in the liraglutide had a resolution of steatohepatitis compared with 9% in the placebo group (*p* = 0.019). Concurrently, only 9% of patients had a progression of liver fibrosis on liraglutide treatment compared with 36% of placebo-treated (*p* = 0.04) [88]. A study in China in 2019, which enrolled 75 patients with MASLD and T2DM with inadequate glycemic control on metformin monotherapy, compared the effect of the addition of liraglutide, insulin glargine, or sitagliptin on LFC. While insulin failed to achieve significant changes, both liraglutide and sitagliptin led to a significant intrahepatic lipid (IHL) reduction in comparison to baseline (15.4% vs. 12.5%, *p* < 0.001), as assessed with magnetic resonance imaging-proton density fat fraction (MRI-PDFF) [90]. Moreover, liraglutide significantly decreased the anthropometric parameters of the patients, namely visceral adipose tissue (VAT) and body weight, compared with baseline [90]. In another double-blind, placebo-controlled RCT among patients with MASLD and T2DM, liraglutide administration was not associated with a significant decrease in LFC (liraglutide: from 18.1% to 120%; placebo: from 18.4% to 14.7%); however, it led to greater body weight and subcutaneous adipose tissue (SAT) loss compared with placebo [89]. In another study in China among patients with T2DM, the combination of metformin with either liraglutide or insulin glargine was associated with a greater reduction in LFC and abdominal adipose tissue when compared to placebo. However, in a head-to-head comparison between the two active groups, the reduction in LFC was not significantly different (−6.3% versus −3.4% in the insulin group) [91]. Khoo et al. compared liraglutide with lifestyle modification, including a supervised diet and exercise program for a period of 26 weeks in 30 obese individuals with MASLD. Although both interventions were similarly effective for body weight loss and LFC reduction at 26 weeks, six months after the discontinuation of both interventions, these beneficial results were not sustained in the liraglutide group in contrast with the lifestyle modification group [92]. An open-label RCT, which enrolled 60 T2DM patients with MASLD and compared the addition of either liraglutide or pioglitazone to the usual care, demonstrated that the decrease in LFC was higher in the liraglutide group [93].

As for the adverse events, in most of the aforementioned studies, mild-to-moderate gastrointestinal events were more commonly observed in patients on liraglutide treatment, including diarrhea, nausea, abdominal discomfort, constipation, and loss of appetite [88,90,91,92,93].

### 4.2. Exenatide

An RCT in France in 2016 recruited 44 obese patients with inadequately controlled T2DM on oral antidiabetic agents, 95% of which had MASLD, and compared exenatide to the standard of care based on local guidelines (Haute Autorité de Santé—National Authority for Health of France). In the control group, antidiabetic treatment was intensified by adding glimepiride or increasing the sulfonylurea dose and, if needed, by adding basal insulin. Exenatide treatment resulted in a significant reduction in LFC compared with the control group (−23.8% versus +12.5%, *p* = 0.007), attributed mainly to the significant decrease in body weight, which was only observed in the exenatide group [94]. Four years later, in 2020, Liu et al. compared exenatide to insulin glargine as a therapeutic approach in patients with newly diagnosed T2DM and MASLD. Although both approaches led to a similar reduction in LFC, exenatide induced greater body weight loss (−5.00 kg vs. −1.25 kg, *p* < 0.001) and a larger decrease in visceral adiposity (waist circumference: −7.03 cm vs. −2.63 cm, *p* < 0.001), liver enzymes, and Fibrosis-4 (FIB-4) index. Notably, the rate of adverse events did not differ significantly between the two groups [95].

### 4.3. Dulaglutide

The effect of dulaglutide on MASLD was investigated in 2020 by Kuchay et al. with an open-label RCT among T2DM patients with MASLD. When compared to the usual care, dulaglutide’s addition to the standard treatment resulted in a 2.6-fold greater reduction in LFC, as well as in improvement in serum γ-glutamyl transferase (GGT) levels. However, the alterations in liver stiffness assessed by Fibroscan, as well as those in the serum AST and ALT levels, were not significant. No serious drug-related events were observed; however, three patients in the dulaglutide group reported upper gastrointestinal symptoms and discontinued the medication [96]. There are some ongoing RCTs that investigate further the effect of dulaglutide on MASH or MASLD (NCT03648554, NCT05140694) [38].

### 4.4. Semaglutide

A multicenter, double-blind, placebo-controlled RCT in 2020 recruited 320 patients with MASH and fibrosis to receive either semaglutide 0.4 mg once daily or placebo for 72 weeks. At the end of the study, 59% of the patients in the semaglutide group had resolution of MASH without the worsening of fibrosis as compared to 17% of their counterparts in the placebo group (*p* < 0.001). However, the proportion of patients with improvement in liver fibrosis was not significantly different between groups [97]. In another placebo-controlled trial among 67 patients with MASLD, semaglutide was associated with a greater reduction in steatosis as compared to placebo at week 24 (−36% vs. −9%, *p* < 0.001), week 48 (−58% vs. −11%, *p* < 0.001), and week 72 (−58% vs. −17%, *p* < 0.001). Nonetheless, no significant difference in liver stiffness between the two groups was reported [98]. In a recent multicenter trial, 71 overweight patients with MASH-related cirrhosis confirmed with biopsy, were randomly assigned to receive either semaglutide 2.4 mg weekly or placebo for 48 weeks, and a follow-up liver biopsy was performed to assess the outcome. At 48 weeks, the percentage of patients who achieved liver fibrosis improvement with no worsening of MASH did not markedly differ between the two groups. The proportion of patients with MASH resolution was also not significantly different between groups [99]. In 2022, an open-label RCT, which enrolled 108 patients with MASH, compared semaglutide monotherapy to various combinations of semaglutide and/or cilofexor, a farnesoid X receptor agonist, and/or firsocostat, an acetyl-coenzyme A carboxylase inhibitor. The combination therapies seemed to be more effective in the reduction of LFC and serum liver enzymes than semaglutide alone, while the number and severity of adverse effects did not differ remarkably between the groups [100]. Notably, while similar rates of adverse events were reported by Loomba et al. [99], some placebo-controlled RCTs observed a higher incidence of gastrointestinal events in the semaglutide group, including mainly diarrhea, nausea, and loss of appetite [97,98]. Semaglutide is currently being thoroughly investigated for its potential benefit on patients with MASH in ongoing RCTs (NCT04822181, NCT05016882, NCT04971785, NCT04639414) [38]. Notably, the administration of once-weekly subcutaneous semaglutide has already been approved for the treatment of obesity [101].

The aforementioned beneficial effects of GLP-1RA on MASLD could be enhanced with the combination of other incretin hormones.

## 5. Combined Incretin Receptor Agonism for MASLD and MASLD-Related Complications

Tirzepatide is a dual agonist of GIP and GLP-1 receptors that showed superiority compared with insulin degludec in the open-label, parallel-group, phase 3 SURPASS-3 RCT, with greater glycemic and bodyweight control (Table 2) [102]. In this multicenter, multinational study, all doses of weekly tirzepatide (5, 10, or 15 mg) significantly reduced BMI, triglycerides, and VLDL cholesterol, as well as blood pressure. Participants in the tirzepatide group also showed a significant decrease in ALT and AST serum levels [102]. Besides steatosis, GLP-1/GIP RAs ameliorated MASH by reinforcing hepatic insulin sensitivity, enhancing triglyceride lipolysis, and restricting the supply of free fatty acids in the liver [103]. In the SURPASS-3 study, the pooled tirzepatide group (10 mg and 15 mg) exhibited a more prominent LFC reduction compared with the insulin degludec group (−8.09% vs. −3.38%, *p* < 0.0001) [102]. Another post hoc analysis compared tirzepatide to placebo or dulaglutide and confirmed a greater ALT (−6.8 units/L in tirzepatide vs. −6.4 units/L in the dulaglutide group, *p* < 0.01), AST (*p* < 0.033, with no significant difference compared with placebo or dulaglutide), K-18 (−135.2 units/L in the tirzepatide group vs. −31.3 in the placebo group, *p* < 0.015), and pro-C3 (−2.1 ng/mL in the tirzepatide vs. −0.6 in the placebo group, *p* = 0.041) reduction at 26 weeks vs. baseline [104]. The improvement of total adiponectin was also observed in the tirzepatide group compared with dulaglutide (0.9 mg/dl vs. 0.3 mg/dl, respectively, *p* < 0.001), possibly attributed to greater weight loss [104]. Of interest, tirzepatide at once-weekly administration has already been approved for the treatment of obesity [105].

The beneficial effects of dual incretin agonists were verified by another randomized, double-blind, placebo-controlled phase 2b study, in which cotadutide, a dual GLP-1 and GCG RA, was evaluated for its hepatic effect in obese patients with T2DM [106]. Patients receiving once daily cotadutide (100, 200, or 300 mg) compared with the placebo group achieved greater reductions in AST (−1.77%, −6.22%, −9.14%, and 5.65%, respectively, *p* < 0.009), ALT (−7.52%, −12.01%, −14.15%, and 0.93%, respectively, *p* < 0.009), PRO-C3 (−0.38% in the 300 mg cotadutide group vs. 13.04 in the placebo group, *p* = 0.0034), total and LDL cholesterol, triglycerides, and GGT levels, as well as in fatty liver index (−8.08, −6.73, −8.18, −1.62 respectively, *p* < 0.001) after 54 weeks of treatment [106]. Interestingly, the aminotransferase level decrease was not attributed exclusively to weight loss, as weight loss observed in the liraglutide group was similar to that in the cotadutide group, suggesting the glucagon-independent hepatic activity of cotadutide, whereas significant weight loss was observed in the cotadutide group [106].

Pemvidutide is also a dual GLP-1/GCG RA that has been recently evaluated as a component in the treatment of MASH and obesity [107]. In a 24-week multicenter, randomized, double-blind, placebo-controlled trial, patients treated with weekly subcutaneous injections of 1.2, 1.8, or 2.4 mg of pemvidutide exhibited a significant decrease in LFC at 24 weeks compared with the placebo group (−56.3%, −75.2%, −76.4%, −14%, respectively). Furthermore, dose-dependent reductions in ALT levels (−13.3 IU/L, −13.7 IU/L, −15.2 IU/L, −2.2 IU/L, respectively) and body weight were indicated [107]. Consistently, DD01, a dual GLP-1/GCG RA, was evaluated in a phase 1 study, with promising results in hepatic steatosis by reducing LFC by ≥30%, which was assessed using MRI-PDFF. Over the four-week period of the study, patients treated with DD01 had a mean LFC reduction of 52% versus 2.8% in the placebo group [108].

An additional recent phase II randomized, double-blind, parallel-group, placebo-controlled study assessed the effects on HbA1c and body weight of multiple rising doses of survodutide, another dual GLP-1/GCG RA, compared to placebo and semaglutide in T2DM patients [112]. The decrease in HbA1c from baseline was significantly greater for all survodutide dosing groups compared with placebo, while the mean reduction in HbA1c was similar between low-dose survodutide and semaglutide. Of note, survodutide induced a dose-dependent reduction in body weight up to −8.7% compared with baseline, while survodutide ≥ 1.8 mg once weekly provoked higher weight loss than semaglutide 1 mg once weekly after 16 weeks [112]. Nonetheless, treatment with survodutide was not related to remarkable reductions in MASH-related parameters, namely Fibrosis (FIB)-4 score, APRI score and NAFLD fibrosis score compared to placebo. The levels of Pro-C3, a fibrogenic biomarker, substantially decreased from baseline in all active survodutide and semaglutide treatment groups compared with placebo [112].

Given the promising results of dual incretin agonists, the use of triple incretin agonists has been assessed for the treatment of MASLD/MASH. In a phase 1b/2a randomized, double-blind, placebo-controlled study, the weekly administration of HM15211 (efocipegtrutide), a GLP-1/GCG/GIP triple incretin agonist, was assessed in 66 non-T2DM patients with biopsy-proven MASH [109]. Treatment with HM15211 significantly reduced LFC placebo in a dose-dependent manner (mean relative changes from baseline in liver fat at week 12 vs. baseline: −19.6% for 0.01 mg/kg, −36% for 0.02 mg/kg, −38% for 0.04 mg/kg, −59.3% for 0.06 mg/kg, and −5.7% for the placebo group) [109]. Furthermore, HM15211 reduced body weight across all treatment dose groups compared with the placebo arm [(placebo-corrected % reduction of body weight was −1.9%, −3.4%, −2.1%, −3.8%, and −5.1%) at week 12 vs. baseline in 0.01 to 0.08 mg/kg dose cohorts, respectively, *p* < 0.05)] [109]. 

In another 48-week phase 2 study including obese patients, weekly triple hormone (GLP-1/GIP/GCG) RA retatrutide (RETA: LY3437943) was investigated for obesity treatment in 338 obese patients, and 98 of those also suffered from MASLD [111]. The mean relative LFC was remarkably decreased in comparison to placebo at 24 and 48 weeks of treatment vs. baseline [change from baseline at 24 weeks was −42.9% (1 mg), −57.0% (4 mg), −81.4% (8 mg), −82.4% (12 mg), and +0.3% (placebo), and at 48 weeks, it was −51.3% (1 mg), −59.0% (4 mg), −81.7% (8 mg), −86.0% (12 mg), and −4.6% (placebo), all *p* < 0.001 vs. placebo] [111]. Importantly, RETA doses ≥4 mg resulted in increased insulin sensitivity and adiponectin serum levels as well as reduced concentration of triglyceride levels, leptin, and fibroblast growth factor (FGF)-21 compared with placebo. Finally, RETA ≥ 4 mg induced a remarkable placebo-controlled increase in beta-hydroxybutyrate (from 78.0% to 181.2%), a marker of fatty acid oxidation [111], while intriguingly, serum values of ALT and AST did not change consistently versus placebo [111].

## 6. Closing Remarks and Future Perspectives

Despite the use of incretin agonists in MASLD/MASH seems an appealing therapeutic opportunity, the beneficial effects of GLP-1/GIP combined therapy are accompanied by some concerns. Firstly, we shall point out that the weight loss provoked by combined GLP-1/GIP agonists can inevitably cause skeletal muscle loss, particularly in patients who already have skeletal muscle dysfunction, sarcopenia, or sarcopenic obesity, which is a quite common feature in MASLD patients [113,114]. A recent study of 40 patients with T2DM evaluated the effect of a 6-month administration of subcutaneous semaglutide on body composition and shed more light on that issue [115]. At 6 months, semaglutide provoked an average weight loss of 10 kg predominantly due to a decline in fat mass and visceral adipose tissue and, in a small proportion, due to a reduction in skeletal muscle mass [115]. However, importantly, that small reduction in fat-free mass was not associated with a reduction in muscle strength, as assessed by a phase-sensitive bioimpedance analyzer. That effect could be mitigated by advising MASLD patients to increase their physical exercise in the form of both aerobic exercise and resistance training in order to minimize the potential harmful effect on skeletal muscle strength and the development of frailty, which is a rapidly recognized feature in patients with MASLD [116,117]. In this setting, specific pharmacological agents for preserving lean muscle mass have been proposed by the literature, and they include urocortin 2 and urocortin 3, testosterone, growth hormone, and activin type II receptor inhibitors [118,119]. Furthermore, MASLD as a multisystemic disease is strongly related to cardiovascular events, while the MASH fibrosis stage is an independent predictor of incident cardiovascular disease [120]. To this end, GLP-1 agonists have already shown beneficial effects on cardiovascular events and are now recommended for the treatment of T2DM, especially when complicated by atherosclerotic CV disease [121,122,123]. Regarding GIP agonists, controversial data have been derived from animal studies with respect to the association between CVD events and tirzepatide administration, while a human observation study demonstrated that physiologically increased fasting GIP levels were associated with increased carotid intima–media thickness among the elderly population [124]. A prespecified meta-analysis including seven RCTs from the SURPASS program showed that treatment once weekly with tirzepatide at any dose (5 mg, 10 mg, and 15 mg) for up to 104 weeks was not associated with higher risk for major CVD events, including cardiovascular death, myocardial infarction, stroke, and hospitalized unstable angina in patients with T2DM [125]. The ongoing SURPASS-CVOT (NCT04255433) study, which includes a total population of more than 13,000 patients with T2DM and established atherosclerotic CV disease, will provide strong evidence on the CV safety profile of tirzepatide as compared to dulaglutide over a maximum follow-up period of 54 months [126].

Most of the reported adverse events related to incretin hormone therapy are gastrointestinal-related, such as nausea, vomiting, diarrhea, and constipation [127,128]. These are presumably linked to the direct effect of incretins on brain-provoking delayed gastric emptying [129,130]. However, most of those events are more prevalent in the first few months and then are generally well-tolerated; there is a concern about the long-term adherence and tolerance of patients to that medication. To this end, the gradual up-titration of incretin-based treatment is highly recommended since the rate of dose escalation seems to be strongly related to the frequency and severity of gastrointestinal symptoms. In addition, the subcutaneous administration of those drugs may also pose an additional challenge for long-term use. The oral formulation of these medications seems an appealing option to maximize patients’ compliance [131]; however, the bioavailability of the oral versions needs further evaluation.

Interestingly, there is an ongoing debate on whether GIP agonism or antagonism is the appropriate way to engage GIP receptors [127,132]. Yang et al. [133], by utilizing a GIPR antagonist based on human and mouse GIP sequences, showed that this peptide blocked GIP-induced insulin secretion and the subsequent reduction in glucose levels, while its co-administration with semaglutide provoked additive weight loss than semaglutide alone in a DIO mouse model. The greater weight loss induced by combined GLP-1RA and GIPR antagonist has also been validated in other animal studies [134,135,136]. The administration of a mouse anti-murine anti-GIPR antibody in both mice and obese nonhuman primates was associated with reduced food intake, resting respiratory exchange ratio, and body weight gain [134]. Moreover, the GIPR antibody-treated animals showed reduced fasting blood glucose and insulin levels, as well as reduced WAT mass, macrophage infiltration in epididymal WAT, and reduced steatosis compared with the control group [134]. A significant study by Killion et al. demonstrated that chronic GIPR agonism led to desensitization of GIPR in primary differentiated adipocytes in vitro and adipose tissue in vivo and acted like a GIPR antagonist that ultimately prevented body weight gain [137]. In line with this, the genetic deletion of GIP(??) and GIPR or the direct ablation of GIP-secreting K cells prevented diet-induced obesity in mouse models [138,139,140]. 

Of interest, evidence suggests that tirzepatide may act as a partial GIP antagonist due to its lower affinity for GIP receptors compared with native GIP [141], especially at high doses, potentially causing receptor downregulation over time. Consistent with that, Amgen presented data from a phase I study in which the AMG133, a dual GLP-1RA and GIPR antagonist, was under development for the treatment of obesity (NCT04478708).

A major source of scientific interest with respect to dual or triple incretin receptor agonists is whether those agents have direct effects on hepatic lipid metabolism and MASH-related inflammation and fibrosis or whether their efficacy is mediated by improvement in body weight and glycemic control. Although some researchers have demonstrated the presence of GLP-1R in the liver [142], some more recent studies with the use of next-generation sequencing have debated that [68,83,127]; however, the overall findings are still inconclusive. Along this line, the effects of GLP-1RAs on fatty acid oxidation and de novo lipogenesis and lipotoxicity seem to be achieved by administrating very high doses at supra-physiological levels and are mediated by non-ligand-receptor interaction but via indirect pleiotropic routes. However, future research is necessary to assess the potential direct weight loss-independent benefits of GLP-1RAs.

Nonetheless, we shall acknowledge that the GCG RA can lead to increased glycogen flux, ameliorated mitochondrial turnover, and decreased lipid content, as shown in the mice model treated with cotadutide [87]. Those effects are mediated by direct liver glucagon receptor signaling. Importantly, the administration of cotadutide resulted in attenuation of liver fibrosis to a greater extent than liraglutide, despite researchers having adjusted the dose of agents to achieve similar body weight reduction in two mice groups [83]. Based on that, other dual GLP-1/GCG RAs are being evaluated in patients with MASLD/MASH in ongoing human clinical studies, such as BI 456,906 in a phase 2b placebo-controlled RCT (NCT04771273) and efinopegdutide in a phase 2a open-label, active-comparator (semaglutide)-controlled RCT (NCT04944992).

Of note, a proportion of patients with T2DM and/or obesity may not respond or respond minimally to GLP-1RA treatment, especially semaglutide [127], indicating a significant limitation. Although the inadequate responses may be linked to the underlying degree of liver inflammation and fibrosis, lifestyle interventions, and compliance to therapy [31], recent studies have demonstrated that the interindividual genetic variability of the expression of GLP-1R may also be implicated [143]. Indeed, some single nucleotide polymorphisms of GLP-1R, particularly rs6923761 [144], may have an impact on the weight and glycemic response to GLP-1R agonism treatment.

## 7. Conclusions

As the rates of MASLD will probably increase in the coming years due to the epidemic spread of obesity and T2DM, the need for the first approved medication for the disease is more imperative than ever, but still unmet. Treatment with GLP-1RAs provokes, via pleiotropic routes, decreased appetite, reduced calorie intake, negative energy balance, and net weight loss. Concurrently, in some RCTs, GLP-1RA treatment significantly reduced LFC compared with placebo or active medication, but in others, it did not. The latest evidence has shown that treatment with dual GLP-1RA/GIP RAs, GLP-1/GCG RAs, or even triple GLP-1/GIP/GCG RAs may be even more effective in the amelioration of MASLD and MASH. Moreover, MASLD is a multisystem disease and not a liver disorder per se, associated with an increased risk of extrahepatic complications, namely CVD [145], T2DM [146], and chronic kidney disease [147], as well as malignancies, including colorectal and breast cancer [148,149]. Therefore, incretin agonists, either alone or in combination, seem an appealing and effective therapeutic opportunity for the disease, as they not only modify liver-related pathways but also act on other tissues affected by MASLD, which have a critical impact on MASLD-related morbidity and mortality. In this direction, the results of the ongoing RCTs are much awaited. Along this line, considering the multiple pathophysiological pathways implicated in the disease, the combination of a GLP-1RA with other agents, such as sodium-glucose cotransporter-2 inhibitor [NCT04639414 and NCT05140694], fibroblast growth factor-21 [NCT05016882], farnesoid X receptor agonist, or Acetyl-CoA carboxylase inhibitor [NCT04971785] for the treatment of MASLD are under investigation. Finally, the demonstration of the safety profile of incretin agents and the evaluation of the long-term compliance of patients to those remain a necessity. Importantly, more data pertinent to the impact of these therapeutic agents on MASLD-related fibrosis, the most determinant factor of clinical outcomes, are needed; this is a great field for ongoing and future research.

## Figures and Tables

**Table 1:** Published phase II or II placebo or active-controlled randomized clinical trials evaluating GLP-1RAs for the treatment of MASLD/MASH.

Author/Ref.	Country/Publication Year	Phase and Design of Study	Study Population	Mean Age, Mean BMI, Gender, T2DM (%)	Intervention; Duration; Assessment	Outcomes
Armstrong et al. [88]	United Kingdom, 2016	Phase 2, double-blind, placebo-controlled	52 overweight patients with biopsy-confirmed MASH	51 yo, 36 kg/m^2^, 60% male, 33% with T2DM	Liraglutide 1.8 mg/day (n = 26) vs. placebo (n = 26); 48 weeks; Liver Biopsy	Greater MASH resolution in the liraglutide group: 39% vs. 9% in the placebo group (*p* = 0.019). Less liver fibrosis progression with liraglutide: 9% vs. 36% in the placebo group (*p* = 0.04).
Bizino et al. [89]	Netherlands, 2020	Phase 2, double-blind, placebo-controlled (sub-analysis of the MGNA VICTORIA study)	49 patients with T2DM	60 yo, 32 kg/m^2^, 59% male, 100% with T2DM	Liraglutide 1.8 mg/day (n = 23) vs. placebo (n = 26); 26 weeks; MRI	LFC reduction was not significantly different between groups; liraglutide was associated with significantly greater body weight and subcutaneous fat reduction
Yan et al. [90]	China, 2019	Phase 2, open-label, active-controlled	75 patients with T2DM and MASLD, with inadequate glycemic control by metformin	44 yo, 30 kg/m^2^, 69% male, 100% with T2DM	Liraglutide 1.8 mg/day (n = 24) vs. insulin glargine 0.2 IU/kg/day (n = 24) vs. sitagliptin 100 mg/day (n = 27) (adds-on metformin); 26 weeks; MRI	When combined with metformin, both sitagliptin and liraglutide but not insulin glargine resulted in a significant decrease in LFC; liraglutide group: from 15.4% [SD 5.6] to 12.5% [SD 6.4] (*p* < 0·001).
Guo et al. [91]	China, 2020	Phase 2, open-label, placebo-controlled	96 patients with T2DM and MASLD with inadequate glycemic control by metformin	52 yo, 29 kg/m^2^, 56% male, 100% with T2DM	Liraglutide 1.8 mg/day (n = 32) vs. insulin glargine once daily (n = 32) vs. placebo (n = 32) (adds-on metformin); 26 weeks; MRI	When combined with metformin, liraglutide significantly reduced steatosis from 26.4% [SD 3.2] to 20.6% [SD 3.9] (*p* < 0·05).
Khoo et al. [92]	Singapore, 2019	Phase 2, open-label, active-controlled	30 patients with obesity and MASLD	41 yo, 33 kg/m^2^, 90% male, 0% with T2DM	Liraglutide 3 mg/day (n = 15) vs. lifestyle modifications: diet and exercise (n = 15); 26 weeks; MRI	Both liraglutide and lifestyle modifications resulted in significant hepatic fat reduction vs. baseline: −7.0% [SD 7.1] and −8.1% [SD 13.2], respectively. These benefits were not sustained in the liraglutide group in a 6-month period.
Zhang et al. [93]	China, 2020	Phase 2, open-label, active-controlled	60 patients with T2DM and MASLD	51 yo, 27 kg/m^2^, 47% male, 100% with T2DM	Liraglutide 1.2 mg/day (n = 30) vs. pioglitazone 30 mg/day (n = 30) (add-on to usual care); 24 weeks; MRI	The addition of liraglutide was associated with a significant reduction in LFC from 24.1% [SD 3.0] to 20.1% [SD 3.8] (*p* < 0·05). This reduction was significantly greater compared with the addition of pioglitazone.
Dutour et al. [94]	France, 2016	Phase 2, open-label, active-controlled	44 patients with obesity and T2DM, with inadequate glycemic control by oral antidiabetic therapy (MASLD in 95% of them)	52 yo, 36 kg/m^2^, 48% male, 100% with T2DM	Exenatide 5–10 μg twice/day (n = 22) vs. reference treatment according to local guidelines (n = 22); 26 weeks; MRI	Exenatide resulted in a significant decrease in hepatic triglyceride content: −23.8% [SD 9.5] vs. +12.5% [SD 9.6] in the placebo group (*p* = 0.007).
Liu et al. [95]	China, 2020	Phase 2, open-label, active-controlled	76 patients with newly diagnosed T2DM and MASLD. Age: 48, BMI: 28, 50% male	48 yo, 28 kg/m^2^, 50% male, 100% with T2DM	Exenatide 5–10 μg twice/day (n = 38) vs. insulin glargine 0.2 IU/kg/day (n = 38); 24 weeks; MRI and Fibroscan	Exenatide and insulin glargine both significantly reduced LFC, but exenatide induced a greater reduction in body weight, visceral adiposity, liver enzymes, and Fibrosis-4 (FIB-4) index.
Kuchay et al. [96]	India, 2020	Phase 2, open-label, active-controlled	64 patients with T2DM and MASLD	47 yo, 30 kg/m^2^, 70% male, 100% with T2DM	Dulaglutide 1.5 mg/week adds-on usual care (n = 32) vs. usual care (n = 32); 24 weeks; MRI and Fibroscan	Addition of dulaglutide resulted in a 2.6-fold greater reduction in LFC and a significant improvement in serum GGT level vs. control group. Changes in liver stiffness on Fibroscan, serum AST, and ALT levels were not significant.
Newsome et al. [97]	Multicenter, 2020	Phase 2, double-blind, placebo-controlled	320 patients with biopsy-confirmed MASH and liver fibrosis of stage F1, F2, or F3	55 yo, 36 kg/m^2^, 41% male, 62% with T2DM	Semaglutide 0.1 mg/day (n = 80) vs. semaglutide 0,2 mg/day (n = 78) vs. semaglutide 0.4 mg/day (n = 82) vs. placebo (n = 80); 72 weeks; liver biopsy	The proportion of patients on semaglutide 0.4 mg/day with resolution of MASH without worsening of fibrosis was significantly higher compared with the placebo group: 59% vs. 17% (*p* < 0.001). Improvement of liver fibrosis was not significantly different between groups.
Flint et al. [98]	Germany, 2021	Phase 1, double-blind, placebo-controlled	67 patients with MASLD (assessed by MRI-PDFF and MRE)	60 yo, >30 kg/m^2^, 70% male, 73% with T2DM	Semaglutide 0·4 mg/day (n = 34) vs. placebo (n = 33); 72 weeks, MRI	In the semaglutide group, hepatic steatosis presented a significantly greater decrease vs. placebo group at week 24 (−36% vs. −9%, *p* < 0.001), week 48 (−58% vs. −11%, *p* < 0.001), and week 72 (−58% vs. −17%, *p* < 0.001); no significant difference between groups was observed in changes of liver stiffness.
Loomba et al. [99]	Multicenter, 2023	Phase 2, double-blind, placebo-controlled	71 patients with biopsy-confirmed MASH-related cirrhosis and BMI ≥ 27 kg/m^2^	60 yo, 35 kg/m^2^, 31% male, 75% with T2DM	Semaglutide 2.4 mg/week (n = 47) vs. placebo (n = 24); 48 weeks, liver biopsy	Neither the proportion of patients with MASH resolution nor the proportion of patients with liver fibrosis improvement without worsening of MASH differed significantly between groups.
Alkhouri et al. [100]	USA, 2022	Phase 2, open-label, active-controlled	108 patients with MASH (assessed by liver biopsy or by MRI-PDFF ≥10% and Fibroscan measured liver stiffness ≥7 kPa)	56 yo, 35 kg/m^2^, 30% male, 55% with T2DM	Semaglutide 2.4 mg/week (n = 21) vs. semaglutide 2.4 mg/week + cilofexor 30 mg/day (n = 22) vs. semaglutide 2.4 mg/week + cilofexor 100 mg/day (n = 22) vs. semaglutide 2.4 mg/week + firsocostat 20 mg/day (n = 22) vs. semaglutide 2.4 mg/week + cilofexor 30 mg/day + firsocostat 20 mg/day (n = 21); 24 weeks; MRI and Fibroscan	Overall, combination therapies resulted in a larger reduction in LFC and greater improvement in liver enzymes and liver fibrosis (as assessed by Fibroscan) than semaglutide alone. When compared to semaglutide monotherapy, the only treatment group with significantly different change in liver steatosis was semaglutide + firsocostat: −11% vs. −8% in the semaglutide monotherapy group (*p* = 0.035).

Abbreviations: ALT, alanine aminotransferase; AST, aspartate aminotransferase; MASLD, metabolic dysfunction-associated steatotic liver disease; MASH, metabolic dysfunction-associated steatohepatitis; MRI, magnetic resonance imaging; PDFF, proton density fat fraction; T2DM, type 2 diabetes mellitus; GGT, gamma-glutamyltransferase.

**Table 2:** Current randomized clinical studies evaluating double or triple incretin receptor agonists for the treatment of MASLD/MASH (or aspects of MASLD in non-MASLD cohorts).

Author/Ref.	Country/Publication Year	Phase and Design of Study	Study Population	Mean Age, Mean BMI, Gender, T2DM (%)	Intervention; Duration; Assessment	Outcomes
Ludvik et al./ Hartman et al. [102,104]	Multinational (13 countries), 2021	Phase 3, randomized, open-label, parallel-group, multicenter	1444 overweight patients with T2DM	57 yo, 33 kg/m^2^, 56% male, 100% with T2DM	Tirzepatide (5, 10, 15 mg) (n = 358, 360, 359) vs. insulin degludec (n = 360); 52 weeks; HbA1c and bodyweight reduction	Greater reduction in HbA1c vs. baseline [1.93%, 2.2%, 2.37% for 5, 10, and 15 mg, respectively (*p* = 0.05)] and body weight and lower risk of hypoglycemia; pooled tirzepatide group (10 mg and 15 mg) induced a greater LFC reduction compared with the insulin degludec group (−8.09% vs. −3.38%, *p* < 0.0001)
Hartman et al. [104]	USA/2020	Phase 2, post-hoc analysis	316 patients with T2DM	57 yo, 32.6 kg/m^2^, 53% male	Tirzepatide (1, 5, 10, 15 mg) (n = 52, n = 55, n = 51, n = 53) vs. dulaglutide (n = 54) or placebo (n = 51); 26 weeks; hepatic dysfunction parameters	Greater decrease in ALT (−6.8 units/L and −6.4 units/L for tirzepatide 10 mg and 15 mg, respectively vs. dulaglutide, *p* < 0.05), K-18 (−135.2 units/L in the tirzepatide 10 mg vs. placebo group, *p* < 0.015) pro-C3 (−2.1 ng/mL in the tirzepatide 15 mg vs. placebo group, *p* = 0.041)
Nahra et al. [106]	Multinational (8 countries), 2021	Phase 2b, double-blind, placebo-controlled	834 patients with T2DM and BMI ≥ 25 kg/m^2^	~56 yo, ~35 kg/m^2^, ~45% male, 100% with T2DM	Cotadutide 100 μg (n = 100), 200 μg (n = 256), or 300 μg (n = 256) vs. placebo (n = 110) or liraglutide 1.8 mg (n = 110); 54 weeks; HbA1c, body weight, hepatic parameters for liver fibrosis	Cotadutide (100, 200, or 300 mg) compared with the placebo group achieved greater reductions in AST (−1.77%, −6.22%, −9.14%, and 5.65%, respectively, *p* < 0.009), ALT (−7.52%, −12.01%, −14.15%, and 0.93%, respectively, *p* < 0.009), PRO-C3 (−0.38% in 300 mg cotadutide group vs. 13.04 in the placebo group, *p* = 0.0034), total and LDL cholesterol, triglycerides, and GGT levels, as well as in fatty liver index (−8.08, −6.73, −8.18, and −1.62, respectively, *p* < 0.001).
Haririson et al. [107]	United States, 2023	Randomized, double-blind, placebo-controlled	94 patients with MASLD	36 kg/m^2^, 29% with T2DM	Pemvidutide 1.2 mg, 1.8 mg, and 2.4 mg vs. placebo; 24 weeks; reduction in LFC, Ct1, ALT, body weight	Pemvidutide reduced LFC at 24 weeks vs. baseline, compared with the placebo group (−56.3%, −75.2%, −76.4%, and −14%, respectively). Dose-dependent reduction in ALT levels (−13.3 IU/L, −13.7 IU/L, −15.2 IU/L, −2.2 IU/L, respectively).
To et al. [108]	United States, 2023	Phase 1	18 overweight/obese patients with T2DM and MASLD	100% with T2DM	DD01 1–80 mg (four once-weekly doses) vs. placebo; 36 days; MRI	Rapid reductions in hepatic steatosis assessed by MRI, HbA1c and greater weight loss. Over the four-week period of the study, patients treated with DD01 had a mean LFC reduction of 52% versus a 2.8% reduction in the placebo group.
Abdelmalek et al. [109,110]	United States, 2020	Phase 1b/2a, multicenter, randomized, placebo-controlled	66 non-diabetic obese patients with MASLD	46 yo; 50% men; mean BMI: 36 kg/m^2^, 0% with T2DM	HM15211 0.01, 0.02, 0.04, 0.06, and 0.08 mg/day vs. placebo; 12 weeks; MRI-PDFF	HM15211 reduced LFC vs. placebo in a dose-dependent manner (mean relative changes from baseline in liver fat at week 12 vs. baseline: −19.6% for 0.01 mg/kg, −36% for 0.02 mg/kg, −38% for 0.04 mg/kg, −59.3% for 0.06 mg/kg, and −5.7% for the placebo group, *p* < 0.05). HM15211 reduced body weight across all treatment dose groups compared with the placebo arm at week 12 vs. baseline [(placebo-corrected % reduction of body weight was −1.9%, −3.4%, −2.1%, −3.8%, and −5.1%) in 0.01 to 0.08 mg/kg dose cohorts, respectively, *p* < 0.05)]
Sanyal et al. [111]	United States, 2023	Phase 2	338 obese MASLD patients	46.6 yo, 38.4 kg/m^2^, 53.1% males, 0% with T2DM	Retatrutide 1, 4, 8, and 12 mg/day vs. placebo; 48 weeks; liver fat change	Retatrutide reduced mean relative LFC in comparison to placebo at 24 and 48 weeks of treatment vs. baseline [change from baseline at 24 weeks was −42.9% (1 mg), −57.0% (4 mg), −81.4% (8 mg), −82.4% (12 mg) and +0.3% (placebo), and at 48 weeks was −51.3% (1 mg), −59.0% (4 mg), −81.7% (8 mg), −86.0% (12 mg) and −4.6% (placebo), all *p* < 0.001 vs. placebo)

Abbreviations: ALT, alanine aminotransferase; AST, aspartate aminotransferase; GGT, gamma-glutamyl transferase; HbA1c, Hemoglobin A1C; LDL, low-density lipoprotein; LFC, liver fat content; MASLD, metabolic dysfunction-associated steatotic liver disease; MASH, metabolic dysfunction-associated steatohepatitis; MRI, magnetic resonance imaging; PDFF, proton density fat fraction; T2DM, type 2 diabetes mellitus.

## Data Availability

No new data were created or analyzed in this study. Data sharing is not applicable to this article.
